# Effects of Rapamycin Treatment on Neurogenesis and Synaptic Reorganization in the Dentate Gyrus after Controlled Cortical Impact Injury in Mice

**DOI:** 10.3389/fnsys.2015.00163

**Published:** 2015-11-27

**Authors:** Corwin R. Butler, Jeffery A. Boychuk, Bret N. Smith

**Affiliations:** ^1^Department of Physiology, College of Medicine, University of KentuckyLexington, KY, USA; ^2^Epilepsy Center, University of KentuckyLexington, KY, USA; ^3^Center for Advanced Translational Stroke Science, University of KentuckyLexington, KY, USA; ^4^Spinal Cord and Brain Injury Research Center (SCoBIRC), University of KentuckyLexington, KY, USA

**Keywords:** epilepsy, trauma, hippocampus, mTOR, dentate granule cell, mossy fiber sprouting, adult neurogenesis, doublecortin

## Abstract

Post-traumatic epilepsy (PTE) is one consequence of traumatic brain injury (TBI). A prominent cell signaling pathway activated in animal models of both TBI and epilepsy is the mammalian target of rapamycin (mTOR). Inhibition of mTOR with rapamycin has shown promise as a potential modulator of epileptogenesis in several animal models of epilepsy, but cellular mechanisms linking mTOR expression and epileptogenesis are unclear. In this study, the role of mTOR in modifying functional hippocampal circuit reorganization after focal TBI induced by controlled cortical impact (CCI) was investigated. Rapamycin (3 or 10 mg/kg), an inhibitor of mTOR signaling, was administered by intraperitoneal injection beginning on the day of injury and continued daily until tissue collection. Relative to controls, rapamycin treatment reduced dentate granule cell area in the hemisphere ipsilateral to the injury two weeks post-injury. Brain injury resulted in a significant increase in doublecortin immunolabeling in the dentate gyrus ipsilateral to the injury, indicating increased neurogenesis shortly after TBI. Rapamycin treatment prevented the increase in doublecortin labeling, with no overall effect on Fluoro-Jade B staining in the ipsilateral hemisphere, suggesting that rapamycin treatment reduced posttraumatic neurogenesis but did not prevent cell loss after injury. At later times post-injury (8–13 weeks), evidence of mossy fiber sprouting and increased recurrent excitation of dentate granule cells was detected, which were attenuated by rapamycin treatment. Rapamycin treatment also diminished seizure prevalence relative to vehicle-treated controls after TBI. Collectively, these results support a role for adult neurogenesis in PTE development and suggest that suppression of epileptogenesis by mTOR inhibition includes effects on post-injury neurogenesis.

## Introduction

Traumatic brain injury (TBI) can result in post-traumatic epilepsy (PTE) in a significant proportion of moderate to severe TBI patients, and PTE accounts for about 20% of symptomatic epilepsies (Caveness et al., 1979; Annegers et al., 1998; Englander et al., 2003). PTE most commonly manifests as neocortical or temporal lobe epilepsy (TLE; Diaz-Arrastia et al., 2000; Hudak et al., 2004). Preventative therapies for PTE have been largely ineffective or have had varying outcomes depending on the type of epilepsy, leaving ~30% of PTE patients intractable to medical therapies (Temkin et al., 1998, 2001; Temkin, 2009). One treatment proposed to prevent PTE in mice is the use of the mammalian target of rapamycin (mTOR) inhibitor rapamycin after injury (Guo et al., 2013). Rapamycin has shown promise in reducing aberrant axonal sprouting and some forms of epileptogenesis, but its effectiveness in preventing seizures in models of acquired epilepsy has been inconsistent (Zeng et al., 2008; Buckmaster and Wen, 2011; Guo et al., 2013; Heng et al., 2013). Studies of rapamycin effects in chemical convulsant models of TLE indicated that rapamycin reduced or eliminated mossy fiber sprouting, but did not prevent spontaneous seizures (Buckmaster and Wen, 2011; Heng et al., 2013). Rapamycin suppressed the development of PTE in mice after controlled cortical impact (CCI) injury, but mossy fiber sprouting recurred after cessation of treatment (Guo et al., 2013). Although mossy fiber sprouting is a hallmark of TLE in animal models and in patients, its causative association with epilepsy development is still controversial and functional outcomes of rapamycin treatment on synaptic reorganization in models of acquired epilepsy are not well described.

Expanding our understanding of how rapamycin treatment exerts its disease-modifying effects in a model of PTE may identify key antiepileptogenic components of mTOR inhibition and guide future treatments and therapeutics for PTE. The mechanism(s) by which mTOR inhibition may alter epileptogenesis, however, are not fully described. Some of the known biochemical and structural cellular effects of increased mTOR signaling include increased protein synthesis, cell growth, and cell proliferation, which may contribute to several outcomes associated with both TBI and TLE, including mossy fiber sprouting, recurrent excitation of dentate granule cells, and enhanced neurogenesis in the dentate gyrus (Buckmaster and Dudek, 1997; Parent and Lowenstein, 1997; Winokur et al., 2004; Parent et al., 2006). Selective genetic upregulation of mTOR activity in newborn granule cells leads to an epilepsy phenotype (Hester and Danzer, 2013), and increased adult neurogenesis has been hypothesized to contribute substantially to epileptogenesis (Parent et al., 2006; Kron et al., 2010). In this study, we investigated cellular, electrophysiological, and disease modifying effects of rapamycin treatment after CCI in mice, a model of PTE (Hunt et al., 2009, 2010, 2011, 2012; Guo et al., 2013). We tested the hypothesis that continual rapamycin treatment after CCI injury reduces post-injury neurogenesis, mossy fiber sprouting, and synaptic reorganization in the dentate gyrus, which may correlate with reduced seizure expression.

## Materials and Methods

### Animals

Six to eight week old (28–32 g) CD-1 mice (*n* = 139; Harlan, Indianapolis, IN, USA) were housed in a 14 h light/10 h dark cycle. Mice were housed for a minimum of 7 days prior to experimentation in the University of Kentucky vivarium and food and water was provided *ad libitum*. All procedures were approved by the University of Kentucky Animal Care and Use Committee and adhered to NIH guidelines for the care and use of animals.

### Traumatic Brain Injury

Mice were subjected to a severe unilateral, cortical contusion injury by CCI, as described previously (Hunt et al., 2009, 2010, 2011, 2012). Briefly, mice were anesthetized by 2% isoflurane inhalation and placed in a stereotaxic frame. The skull was exposed by midline incision, and a ~5 mm craniotomy was made lateral to the sagittal suture and centered between bregma and lambda. The skull cap was removed, taking care to avoid damage to the exposed underlying dura. The contusion device consisted of a computer-controlled, pneumatically driven impactor fitted with a beveled stainless-steel tip 3 mm in diameter (TBI-0310; Precision Systems and Instrumentation, Fairfax, VA, USA). Brain injury was delivered using this device to compress the cortex to a depth of 1.0 mm at a velocity of 3.5 m/s and 500 ms duration. This brain injury model consistently produced a focal cortical lesion. Although there is no direct damage to the hippocampus from the injury, hippocampal evulsion usually occurs (Hunt et al., 2009, 2012). A qualitative postoperative health assessment was performed daily for 4 days after CCI and periodically thereafter.

### Rapamycin Injection

Rapamycin (LC Laboratories, Woburn, MA, USA) was initially dissolved in 100% ethanol (20 mg/ml), stored at −20°C, and diluted in a vehicle solution containing 5% Tween 80, 5% PEG 400, and 4% ethanol (all from Fisher Scientific, Pittsburgh, PA, USA) dissolved in distilled, deionized water immediately before intraperitoneal (i.p.) injection (Guo et al., 2013; Heng et al., 2013). Rapamycin (3 mg/kg or 10 mg/kg) or vehicle was injected i.p. after mice regained consciousness following CCI injury (20–30 min) and the treatment was continued once daily until the day of experimentation. Hippocampal homogenates from mice 24 h after injury indicated an increase in phosphorylated S6 protein (pS6) levels in the ipsilateral hemisphere of CCI-injured mice with vehicle treatment; rapamycin treatment reduced pS6 to sham levels at this post-injury time point (unpublished observation), similar to previous reports (Buckmaster et al., 2009; Zeng et al., 2009; Guo et al., 2013).

### Slice Preparation

Slices used for electrophysiological studies were obtained from mice 8–13 weeks post-CCI. Mice were deeply anesthetized by isoflurane inhalation to effect (i.e., lack of tail pinch response) and decapitated while anesthetized. The brain was removed and placed in ice-cold (2–4°C) oxygenated artificial cerebrospinal fluid (ACSF) containing, in mM: 124 NaCl, 3 KCl, 1.3 CaCl_2_, 26 NaHCO_3_, 1.3 MgCl_2_, 11 glucose and 1.4 NaH_2_PO_4_ equilibrated with 95% O_2–_5% CO_2_ (pH 7.2–7.4). Brains were blocked and glued to a sectioning stage, and 350 μm-thick slices were cut in the coronal or horizontal plane in cold, oxygenated ACSF using a vibrating microtome (Vibratome Series 1000; Technical Products International, St. Louis, MO, USA). The hippocampus was isolated from surrounding tissue, making sure to completely remove the entorhinal cortex. Slices were transferred to a chamber containing oxygenated ACSF at 32–34°C, where they were equilibrated for at least one hour prior to recording. Slices of the septal and temporal hippocampus from the hemispheres ipsilateral and contralateral to CCI injury were used in these experiments and compared to comparable slices from sham-injured mice (i.e., craniotomy, but no impact injury).

### Extracellular Field Potential Recordings

Field potential recordings were obtained from the granule cell layer of the dentate gyrus in horizontal slices. Slices were placed into a submersion type recording chamber (RC21-BW, Warner Instruments, Hamden, CT, USA) on an upright, fixed stage microscope (Olympus BX50WI, Center Valley, PA, USA) and continuously perfused with oxygenated, nominally Mg^2+^ free ACSF containing 30 μM bicuculline to block GABA_A_ receptors, and unmask recurrent excitation (Winokur et al., 2004; Hunt et al., 2009). Extracellular recording electrodes were filled with 1 M NaCl and placed near the apex of the dentate granule cell layer. A concentric bipolar electrode made of platinum-iridium wire (125 μm, FHC Inc., Bowdoinham, ME, USA) was used to apply a single stimulus to the mossy fiber pathway at 0.1 Hz. Stimulus intensity was adjusted to evoke a population response of ~50% maximum amplitude after a single stimulus. Electrical signals were recorded using an Axopatch 200 B amplifier (Axon Instruments, Sunnyvale, CA, USA), low pass filtered at 2–5 kHz, digitized at 20 kHz using a 1322A Digidata (Axon Instruments), and analyzed on a PC computer using pClamp 10.2 (Clampfit, Molecular Devices, Sunnyvale, CA, USA). The number of population spikes following antidromic stimulation of mossy fibers in the hilus was measured as described previously (Hunt et al., 2009).

### Whole Cell Recordings

Coronal hippocampal slices containing the dorsal third of the dentate gyrus were transferred to a recording chamber on an upright, fixed-stage microscope equipped with infrared, differential interference contrast optics (i.e., IR-DIC; Olympus BX50WI), where they were perfused with continuously warmed (32–34°C) ACSF. Recordings were performed from dentate granule cells, which were identified using DIC imaging. Recording pipettes were pulled from borosilicate glass (1.65 mm outer diameter, 0.45 mm inner diameter; King Precision Glass, Claremont, CA, USA) with a P-87 puller (Sutter Instrument, Novato, CA, USA). The intracellular solution contained (in mM): 130 K^+^-gluconate, 1 NaCl, 5 EGTA, 10 HEPES, 1 MgCl_2_, 1 CaCl_2_, 3 KOH, and 2 ATP. Open tip series resistance was 2–5 MOhms. Recordings were obtained using an Axon Multiclamp 700 B amplifier (Molecular Devices), low-pass filtered at 6 kHz, digitized at 20 kHz with a Digidata 1550A (Molecular Devices), and acquired using pClamp 10.5 programs (Clampfit, Molecular Devices). Cells were voltage-clamped at −70 mV for 5–10 min to allow equilibration of pipette and intercellular solutions prior to data collection, after which time whole-cell patch-clamp recordings of spontaneous excitatory postsynaptic currents (sEPSCs) were obtained. All sEPSCs were assessed over a 2–3 min period (>100 events) to assess frequency; amplitude was measured only from unitary events. Although the investigator was not blinded to experimental group for electrophysiological experiments, the investigator was blinded to experimental group for all offline data analyses.

### Timm Staining

After the recording experiment concluded, slices were placed in 0.1 M sodium phosphate buffer containing 0.37% sodium sulfide (pH 7.4) for ~30 min followed by 4% paraformaldehyde in 0.15M sodium phosphate buffer (pH = 7.4) overnight. Slices were then equilibrated in a 30% sucrose solution in phosphate buffered saline (PBS; 0.1 M) overnight, embedded in Optimal Cutting Temperature (OCT) compound (Fisher Scientific), sectioned at 30 μm on a cryostat, rinsed in PBS, mounted on charged slides (Superfrost Plus; Fisher Scientific), and dried on a slide warmer. Sections were subsequently treated as in previous protocols, using Timm stain to reveal mossy fibers and Nissl counterstain to reveal cell bodies (Tauck and Nadler, 1985; Shibley and Smith, 2002; Winokur et al., 2004; Hunt et al., 2009, 2010, 2011, 2012; Bhaskaran and Smith, 2010). To semi-qualitatively assess mossy fiber sprouting after CCI, sections at equivalent positions relative to bregma ipsilateral and contralateral to injury were examined and assigned Timm scores ranging from 0–3, with a score of 0 corresponding to little to no granular staining, 1 indicating moderate Timm staining through the granule cell layer, but not into the inner molecular layer, 2 indicating continuous staining through the granule cell layer with discontinuous puncta in the inner molecular layer, and 3 indicting a continuous band of staining in the inner molecular layer. The scorer was blinded to treatment. Using a modified scoring scale (Hunt et al., 2009, 2010), regions of the dentate gyrus with Timm scores >1 were considered to exhibit mossy fiber sprouting (Tauck and Nadler, 1985; Patrylo and Dudek, 1998; Shibley and Smith, 2002; Hunt et al., 2009, 2010, 2011, 2012). To obtain the Timm score for each animal, each 350 μm slice used for recording was analyzed using two 30 μm sections, ~180 μm apart mounted onto slides. Slices at equivalent hippocampal levels ipsilateral and contralateral to the injury were assessed for each animal. Each blade of the dentate gyrus was assessed independently and the average score of the two blades was given for each slice. The scores from each slice were then averaged per hemisphere per animal to obtain the Timm score for each animal.

### Immunohistochemistry

Mice were perfused transcardially with a 0.15M sodium phosphate buffer followed by 4% paraformaldehyde fixative solution (0.15M sodium phosphate buffer). The brain was removed and placed in fixative overnight and then transferred to a 30% sucrose solution in PBS until the tissue equilibrated. Brains were covered in OCT compound and sectioned serially on a cryostat (−22°C) at 20 μm. Sections (every 6th section in series) were rinsed in Tris-buffered saline (TBS; pH = 7.4) briefly before being mounted onto slides and incubated in a solution containing Triton X-100 (0.3%) and normal goat serum (10%) in TBS for 30 min at room temperature. Sections were then incubated overnight at 4°C with a rabbit primary antibody against doublecortin (DCX; 1:5000; Abcam; Cambridge, MA, USA) in blocking solution (2% normal goat serum; 0.15% Triton X-100; TBS). Sections were rinsed 3 times for 5 min in blocking solution and then incubated for 1 h at room temperature in a goat anti-rabbit secondary antibody (IgG) conjugated to Alexa Flour 488 (IgG; 1:1000; Molecular Probes; Grand Island, NY, USA) in the same blocking solution. Sections were then rinsed 3 times for 5 min with TBS. Slides were covered with Vectashield mounting medium with DAPI (Vector Labs; Burlingame, CA, USA) to image immunofluorescence.

### Dentate Granule Cell Layer Area

In order to measure changes in area of the dentate granule cell layer after CCI, adjacent tissue sections to those used for DCX labeling were stained with cresyl violet (i.e., every 6th section in series). Sections were imaged using a SPOT RT camera (Diagnostic Instruments; Sterling Heights, MI, USA) mounted on an upright microscope (BX-40; Olympus) and dentate granule cell area measurements were made using ImageJ software. Images were taken at 4× and 10× magnification to capture the entire dentate gyrus. After scaling the ImageJ software, the cresyl violet-stained dentate granule cell layer was traced freehand and the area measured. The area measurements were assessed in two different ways. The first was to assess overall changes in dentate granule cell layer area across the hippocampus from approximately −1.22 to −3.52 mm from bregma. In this assessment, measurements from each tissue section were made to obtain the overall average of the dentate granule cell layer area for each hemisphere in each animal. Additionally, this data set was assessed as a function of anatomical location along the septo-temporal hippocampal axis relative to the injury epicenter using a mouse brain atlas (Paxinos and Franklin, 1997).

#### Fluoro-Jade B (FJB) Staining

Protocols used were similar to those reported previously (Hall et al., 2008). In brief, sections (30 μm) from perfused brains were mounted on slides and treated with a solution of 1% NaOH in 80% ethanol for 5 min followed by 70% ethanol (2 min) and distilled water (2 min). Sections were then incubated in a 0.06% permanganate solution for 10 min on a rotating stage, rinsed in distilled water (3 min) and incubated in a 0.0004% solution of FJB (Histo-Chem Inc., Jefferson, AR, USA; 10 min). They were then rinsed in distilled water and air dried before being placed on a 50°C slide warmer for 30 min. They were then placed in xylene for 20 min and coverslipped in permount.

#### Cell Counts

Numbers of DCX-immunolabeled dentate granule cells and FJB-labeled neurons were counted between −1.22 to −3.52 mm from bregma in the upper and lower blade of the dentate granule cell layer at 20× and 40× magnification (Olympus, BX40) by an investigator blinded to animal treatment. For figures, representative images were taken at 4× and 10× magnification to display the whole dentate gyrus. DCX cell counts were normalized to dentate granule cell layer area, as above. The area of the dentate gyrus and hilus was obtained to normalize FJB labeling counts and included the dentate granule cell layer and the hilus inside the region outlined by a line around the outer edge of the granule cell layer and connecting the tips of the dentate granule cell layer with the most proximal point of the CA3 pyramidal cell layer. For each animal, cell density was calculated per section and then averaged across the entire hippocampus to obtain an overall measure of cell density. Additionally, cell density was measured as a function of distance from bregma. For these measurements tissue sections were placed in anatomical order using a mouse brain atlas (Paxinos and Franklin, 1997) and cell density was averaged for each animal at each anatomical location.

#### Seizure Observations

As described previously (Hunt et al., 2009), mice were monitored for behavioral seizures by observation for 6 h per week beginning at 6 weeks post-injury and ending 10 weeks post-injury. Using a modified Racine scale (Racine, 1972) only behavioral seizures at or above a grade 2 (i.e., prolonged freezing and wet dog shakes) and lasting longer than 10 s were counted as behavioral seizures.

## Results

We compared cellular and behavioral outcomes (i.e., FJB staining, DGC area, DCX staining, mossy fiber sprouting, field potential responses, sEPSC frequency, and seizures) in the dentate gyrus of mice from sham-injured, CCI-injured with vehicle treatment, and CCI-injured with rapamycin treatment (3 or 10 mg/kg). For cellular outcomes, each hemisphere was assessed independently for all groups. Initial comparisons between hemispheres from sham-injured mice and the hemisphere contralateral to CCI injury in vehicle- and rapamycin-treated mice indicated there were no differences in the cellular outcomes for these groups. Data from sham and contralateral hemispheres were compared using a One-way ANOVA with Tukey’s *post hoc* analysis, or Kruskal Wallis/Chi-square statistic where necessary, and significance was set at *p* < 0.05. No significant differences were found for any measure between sham-injured mice and those made in the hemisphere contralateral to CCI injury (*p* > 0.05). For each analysis of cellular outcomes, these groups were therefore combined as a single control group for clarity of presentation. Table 1 compares results of measurements from both hemispheres after sham surgery with results from the hemisphere contralateral to CCI injury in mice treated with vehicle or rapamycin.

**Table 1:** **Measures from sham-operated mice (both hemispheres) and the hemisphere contralateral to injury after CCI in mice treated with vehicle or rapamycin**.

Group	FJB + cell density (FJB-positive cells/mm^2^)	DGC area (mm^2^)	DCX + cell density (DCX-positive cells/mm^2^)	Timm score	Recurrent excitation (% slices with secondary depolarization)	sEPSC frequency (Hz)
Sham (contralateral)	N/A	0.130 ± 0.012 (*n* = 7)	774.50 ± 96.37 (*n* = 6)	0.285 ± 0.02	0% (0/5 slices)	N/A
Sham (ipsilateral)	N/A	0.130 ± 0.012 (*n* = 7)	637.42 ± 44.42 (*n* = 6)	0.248 ± 0.047	20% (1/5 slices)	N/A
CCI + vehicle (contralateral)	7.07 ± 1.20 (*n* = 7)	0.133 ± 0.013 (*n* = 8)	957.00 ± 133.99 (*n* = 6)	0.260 ± 0.034	25% (2/8 slices)	0.83 ± 0.11 (*n* = 14)
CCI + Rapa (3 mg/kg; contralateral)	8.33 ± 1.30 (*n* = 7)	0.130 ± 0.013 (*n* = 6)	742.77 ± 142.46 (*n* = 5)	0.333 ± 0.108	20% (2/10 slices)	0.5 ± 0.10 (*n* = 9)
CCI + Rapa (10 mg/kg; contralateral)	7.19 ± 1.32 (*n* = 6)	0.126 ± 0.014 (*n* = 7)	760.86 ± 131.20 (*n* = 6)	0.437 ± 0.091	25% (2/8 slices)	0.81 ± 0.26 (*n* = 12)

*One-way ANOVA with Tukey’s post hoc analysis, or Kruskal Wallis/Chi-square statistic where appropriate, were used to obtain the p-value; p > 0.05 for all analyses*.

### Behavioral Seizure Monitoring

Subsets of mice were monitored for behavioral seizures after severe (1.0 mm depth) unilateral CCI injury from 6–10 weeks post-injury (6 h/week) to qualitatively assess spontaneous seizure development. Consistent with previous reports, four of 10 mice (40%) that received CCI injury and vehicle treatment displayed spontaneous seizures during this period post-injury (Hunt et al., 2009, 2010; Guo et al., 2013). All were S2 seizures with tail stiffness and freezing for more than 30 s. One of twelve mice (8%) in the low-dose (3 mg/kg) rapamycin-treated group and one of 11 mice (9%) in the high-dose (10 mg/kg) rapamycin-treated group were observed to have spontaneous seizures (all category S2). Although numerically lower in the rapamycin treatment groups, and a trend toward reduced seizures was apparent, the prevalence of observed seizures in CCI injured mice with no treatment and CCI injured mice with rapamycin treatment was not significantly different using a Chi-square test of probability (3 mg/kg, *p* = 0.078; 10 mg/kg, *p* = 0.097). These data indicate that mTOR activity may influence seizure development after CCI in some cases, but mTOR inhibition is not sufficient to prevent epileptogenesis after CCI injury in all mice.

### Fluoro-Jade B (FJB) Labeling

**Figure 1:**
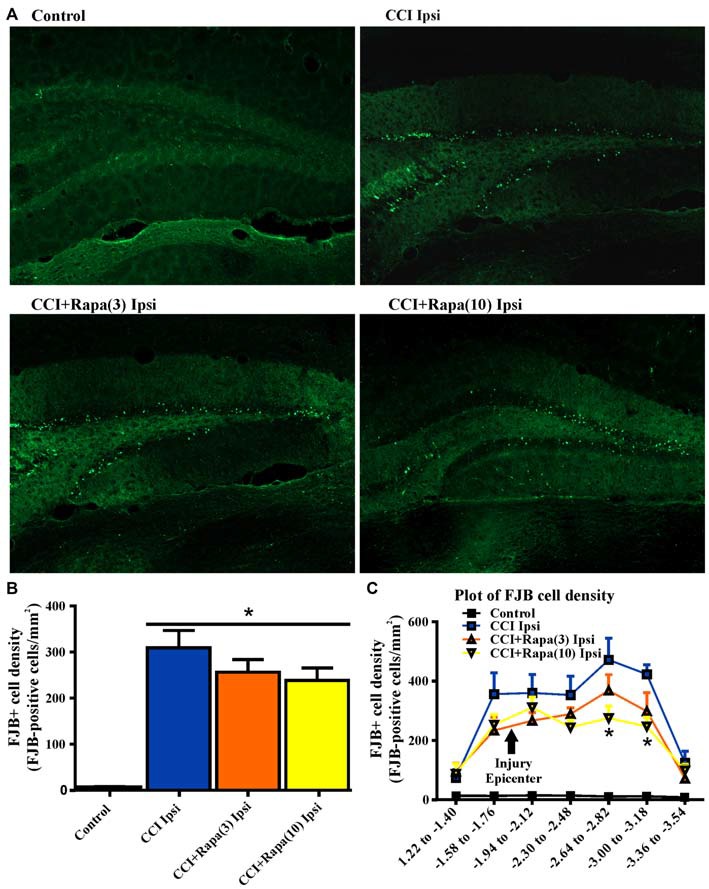
**Fluoro-Jade B labeling in dentate gyrus 3 days after control treatment, CCI or CCI with daily rapamycin administration. (A)** Representative images of Fluoro-Jade B (FJB) labeling from four different groups: control, ipsilateral to CCI injury + vehicle (CCI Ipsi), ipsilateral to CCI injury + rapamycin at 3 mg/kg (CCI + Rapa(3) Ipsi), and ipsilateral to CCI injury + rapamycin at 10 mg/kg (CCI + Rapa(10) Ipsi). **(B)** Mean FJB labeling in control, CCI Ipsi, CCI + Rapa(3) Ipsi, and CCI + Rapa(10) Ipsi groups. FJB-positive cell density (cells/mm^2^) was averaged across coronal slices from −1.22 to −3.52 mm from bregma. All CCI ipsilateral hemispheres exhibited increased FJB-positive cell density relative to controls. No significant difference was observed after CCI injury between vehicle- and rapamycin-treated mice. **(C)** FJB-positive cells/mm^2^ as a function of distance from bregma along the septo-temporal axis of hippocampus. The CCI + Rapa(10) group exhibited reduced FJB-positive cell density only in a limited region of posterior hippocampus relative to CCI + vehicle treatment. Error bars indicate SEM; *p < 0.05.

Regional cell loss (i.e., dentate gyrus, hilus, and CA3 pyramids) in the hippocampus is a common feature after TBI (Lowenstein et al., 1992; Hicks et al., 1993; Smith et al., 1995). The role of mTOR in neuronal death and survival after TBI has been controversial (Guo et al., 2013; Tanaka et al., 2013). This is likely due to the complex role mTOR plays in the balance of autophagy and apoptosis after injury. Based on previous reports, FJB staining in the ipsilateral hemisphere peaks following CCI injury in the first 3 days after injury (Anderson et al., 2005). We therefore measured FJB staining at 72 h post-CCI injury to evaluate this peak FJB staining. Representative images of FJB stained sections from CCI-injured mice treated with vehicle, low-dose rapamycin (3 mg/kg), and high-dose rapamycin (10 mg/kg) in hemispheres ipsilateral to the injury are shown in Figure 1A. The granule cell layer and hilus of mice with CCI injury + vehicle (309.1 ± 37.8 FJB-positive cells/mm^2^; *n* = 7 mice), CCI + low-dose rapamycin (256.6 ± 27.3 FJB-positive cells/mm^2^; *n* = 6 mice), and CCI + high-dose rapamycin (238.6 ± 27.0 FJB-positive cells/mm^2^; *n* = 7 mice) treatment displayed significantly more FJB-labeled cells ipsilateral to injury than sham-operated, vehicle-treated controls (7.38 ± 0.73 FJB-positive cells/mm^2^; *n* = 20; one-way ANOVA; (*F*_(3,37)_ = 66.58, Tukey’s; *p* < 0.0001; Figure 1B). There was no difference in the density of FJB-labeled cells between the ipsilateral hemispheres of vehicle- or rapamycin-treated mice after CCI injury (*p* > 0.05).

Previously, rapamycin treatment was reported to reduce FJB staining in one region of the dentate gyrus (Guo et al., 2013), so we investigated FJB staining throughout the septo-temporal dentate gyrus and hilus at 180 μm intervals. We found only two intervals in which FJB staining in CCI-injured mice treated with high dose rapamycin was reduced in the ipsilateral hemisphere relative to CCI + vehicle (Figure 1C). These locations were between −2.62 to −3.18 mm from bregma, which is ~1 mm posterior to injury epicenter (Figure 1C). There was no significant reduction in FJB staining at any other location along the hippocampal axis after CCI injury in rapamycin-treated mice and FJB labeling was significantly increased relative to controls at all septo-temporal locations.

#### Dentate Granule Cell Layer Area

**Figure 2:**
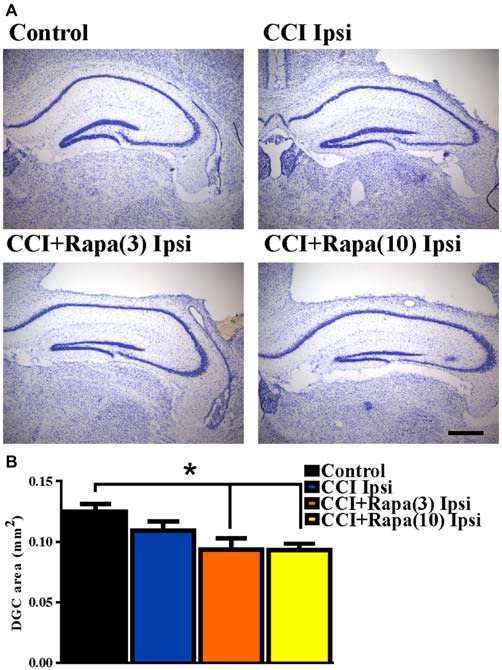
**Dentate granule cell area 14 days after injury in mice from control, CCI-injured, and CCI-injured with rapamycin treatment. (A)** Representative images of Nissl stained sections ipsilateral to injury from the four different treatment groups: sham-operated control, CCI injury + vehicle (CCI Ipsi), CCI injury + 3 mg/kg rapamycin (CCI + Rapa(3) Ipsi), and CCI injury + 10 mg/kg rapamycin (CCI + Rapa(10) Ipsi). Hippocampal sections were from similar anterior-posterior coordinates. **(B)** Mean dentate granule cell area from Nissl stained sections in control, CCI Ipsi, CCI + Rapa(3) Ipsi, and CCI + Rapa(10) Ipsi groups. Scale bar indicates 0.5 mm. Error bars indicate SEM; *p < 0.05.

Both cell death and cell proliferation are common features in the dentate granule cell layer following injuries such as TBI and seizures (Parent et al., 1997; Rola et al., 2006; Carlson et al., 2014). After TBI in rodents, a reduction in dentate granule cell count or area has been observed early (48 h) after injury (Smith et al., 1995). This reduction persists for up to 7 days (Wiltgen et al., 2009) and is alleviated by 2 weeks post-injury (Grady et al., 2003), suggesting cell proliferation compensates for early cell loss after injury. To test the effect of rapamycin on dentate granule cell layer thickness at a time point corresponding to its restoration after CCI injury, dentate granule cell layer area was measured 14 days after injury in Nissl stained sections from control (0.125 ± 0.006 mm^2^; *n* = 35; Figure 2B), CCI with vehicle (0.110 ± 0.009 mm^2^; *n* = 8; Figure 2B), CCI with low-dose rapamycin treatment (0.094 ± 0.010 mm^2^; *n* = 6; Figure 2B), and CCI with high-dose rapamycin treatment (0.093 ± 0.005 mm^2^; *n* = 7; Figure 2B). Representative images of Nissl stained sections are shown in Figure 2A. Rapamycin treatment resulted in a significant reduction of DGC layer area ipsilateral to the injury relative to control (One-Way ANOVA; *F*_(3,41)_ = 6.476, Tukey’s; *p* = 0.0003; Figure 2B). There was a trend toward decreased granule cell layer area after CCI, but a significant change was not detected (*p* = 0.0846).

### Doublecortin (DCX) Immunolabeling

**Figure 3:**
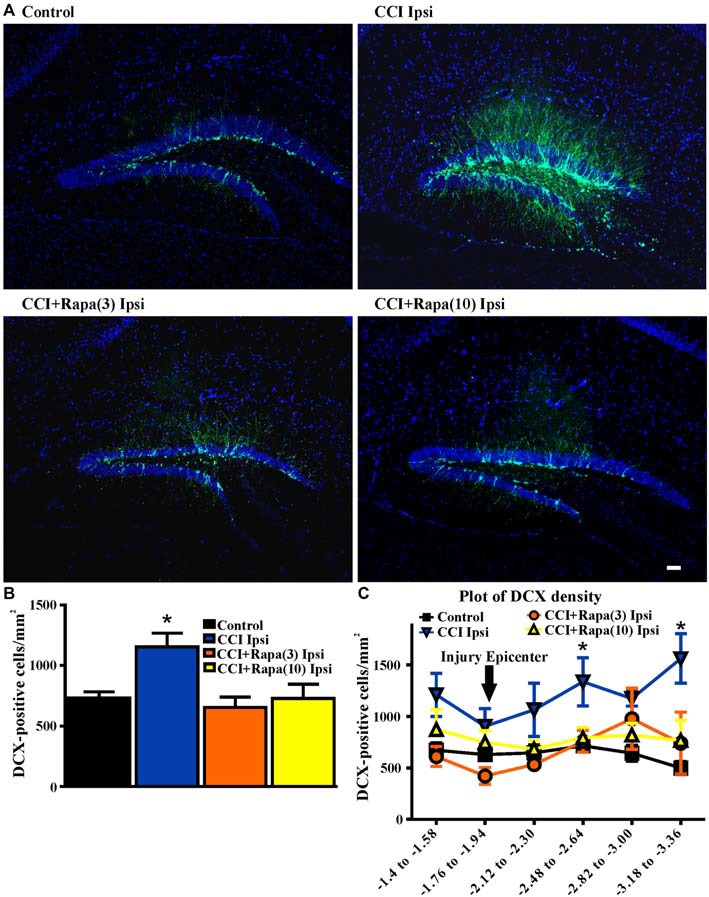
**Doublecortin (DCX) immunolabeling in dentate gyrus 14 days after injury in mice from control, CCI-injured, and CCI-injured with rapamycin groups. (A)** Representative images of DCX expression from four treatment groups: control, CCI Ipsi, CCI + Rapa(3) Ipsi, and CCI + Rapa(10) Ipsi. **(B)** Mean DCX expression in control, CCI Ipsi, CCI + Rapa(3) Ipsi, and CCI + Rapa(10) Ipsi groups. Ipsilateral CCI exhibited greater DCX-positive cell density compared to controls. The injury-induced increase in DCX-positive cell density in CCI ipsi mice was not observed in either CCI + Rapa treatment group. **(C)** DCX-positive cells/mm^2^ as a function of distance from bregma along septo-temporal axis of hippocampus. Scale bar indicates 0.1 mm. Error bars indicate SEM; *p < 0.05.

Shortly after CCI injury there is an initial decrease in DCX expression ipsilateral to injury, but from days 7–14 after injury an increase in DCX expression ipsilateral to the injury has been reported (Dash et al., 2001; Rola et al., 2006; Barha et al., 2011). This proliferation of newborn dentate granule cells after injury or status epilepticus has been proposed to contribute substantially to epileptogenesis (Parent et al., 2006; Hester and Danzer, 2013; Lasarge et al., 2015). Therefore, DCX expression was examined 14 days after injury in control mice (*n* = 29), vehicle-treated CCI-injured mice (*n* = 6), CCI-injured mice with low-dose rapamycin treatment (*n* = 5), and CCI-injured mice with high-dose rapamycin treatment (*n* = 6). Figure 3A shows representative images of DCX staining from the dentate gyrus ipsilateral to the injury (or sham surgery) in these groups. In the ipsilateral hemisphere, a significant increase in DCX expression was observed in vehicle-treated CCI-injured mice relative to controls (Control: 730.04 ± 51.71 DCX-positive cells/mm^2^; CCI: 1154.15 ± 114 DCX-positive cells/mm^2^; One Way ANOVA; *F*_(3,39)_ = 4.838, Tukey’s; *p* = 0.0059; Figure 3B). The relative increase in DCX expression after CCI was observed up to ~1.5 mm temporal to the injury epicenter (Figure 3C). Rapamycin treatment after CCI injury significantly reduced DCX expression to levels similar to control (rapamycin 3 mg/kg: 653.94 ± 85.99 DCX-positive cells/mm^2^, *p* = 0.6098 vs. control; rapamycin 10 mg/kg: 728.51 ± 117.2 DCX-positive cells/mm^2^, *p* = 0.8848 vs. control; Figure 3B). These results are consistent with an inhibitory effect of rapamycin treatment on post-injury neurogenesis.

### Timm Staining

**Figure 4:**
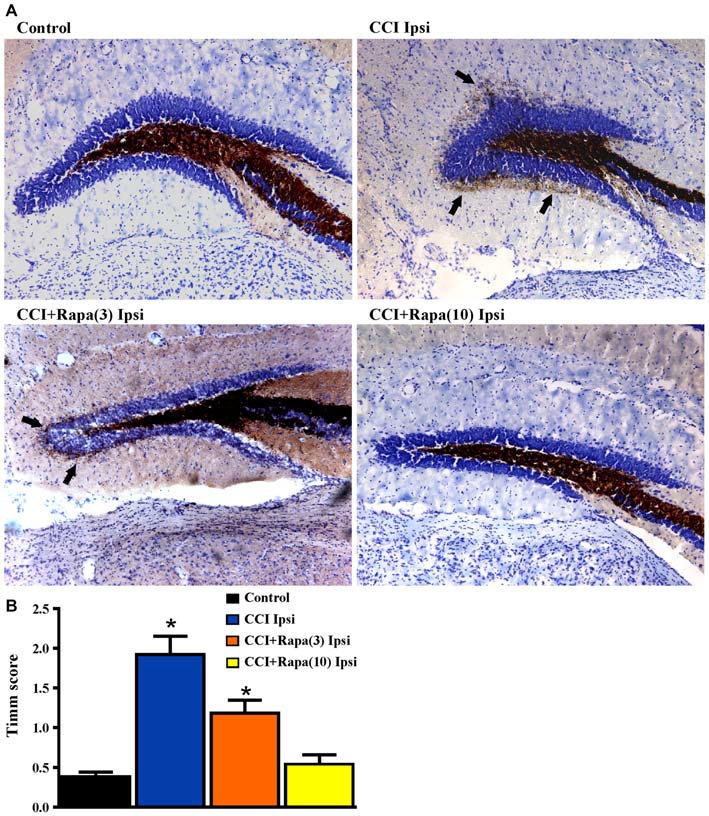
**Timm staining in the dentate gyrus 8–13 weeks post-injury from sham-operated control, CCI injured, and CCI-injured with rapamycin treatment groups. (A)** Representative images of Timm staining from the four different groups: control, CCI Ipsi, CCI + Rapa(3) Ipsi, and CCI + Rapa(10) Ipsi. **(B)** Mean Timm scores in control, CCI Ipsi, CCI + Rapa(3) Ipsi, and CCI + Rapa(10) Ipsi groups. The injured hemisphere of both CCI Ipsi and CCI + Rapa(3) Ipsi groups exhibited higher Timm scores relative to the control group. Mice receiving rapamycin treatment (10 mg/kg) after CCI had Timm scores similar to controls. Error bars indicate SEM; *p < 0.05.

Several weeks after CCI, there is an increase in Timm staining in the inner molecular layer of the dentate gyrus ipsilateral to the injury relative to the contralateral hemisphere or in sham-treated mice (Hunt et al., 2009, 2010, 2011, 2012; Guo et al., 2013). Rapamycin treatment for 4 weeks post-injury reduced Timm staining five weeks after CCI (Guo et al., 2013). However, as with studies done in the pilocarpine-induced status epilepticus model of TLE (Buckmaster et al., 2009), mossy fiber sprouting recurred after cessation of treatment. To assess the effects of continuous rapamycin treatment on mossy fiber sprouting after injury, Timm staining was examined in control mice and in CCI-injured mice treated daily for 8–13 weeks with rapamycin or vehicle. Slices used for extracellular field potential recordings, as well as mice perfused for histology, were used for Timm staining measurements. There was no significant difference between the contralateral hemispheres of any of the groups (all exhibited Timm scores < 1; Table 1). Figure 4A shows representative images of Timm stained sections ipsilateral to the injury from control, CCI + vehicle, CCI + rapamycin (3 mg/kg), and CCI + rapamycin (10 mg/kg) treated mice. In vehicle-treated mice, Timm scores in hemispheres ipsilateral to CCI injury were increased relative to control hemispheres (control: 0.364 ± 0.05, *n* = 31; vehicle + CCI: 2.335 ± 0.300, *n* = 12; Kruskal Wallis stat = 41.41, *p* < 0.0001; Figure 4B). In low-dose rapamycin-treated mice, Timm scores were reduced (1.275 ± 0.315, *n* = 12, Figure 4B) ipsilateral to the injury relative to vehicle-treated mice after CCI injury (*p* = 0.025), but remained greater than in controls (*p* < 0.0001). Mossy fiber sprouting was not different from controls in the high-dose rapamycin treatment group (0.55 ± 0.09, *n* = 11; Kruskal Wallis stat = 16.41; *p* = 0.145 vs. control; Figure 4B). Although the average mossy fiber sprouting score was reduced in mice that received rapamycin treatment, localized areas of mossy fiber sprouting into the inner molecular layer were always observed in the dentate gyrus of mice that expressed spontaneous behavioral seizures. These data indicated that continual rapamycin treatment reduced mossy fiber sprouting after CCI injury and this reduction was maintained during treatment for up to 12 weeks.

#### Network Excitability in Dentate Gyrus

**Figure 5:**
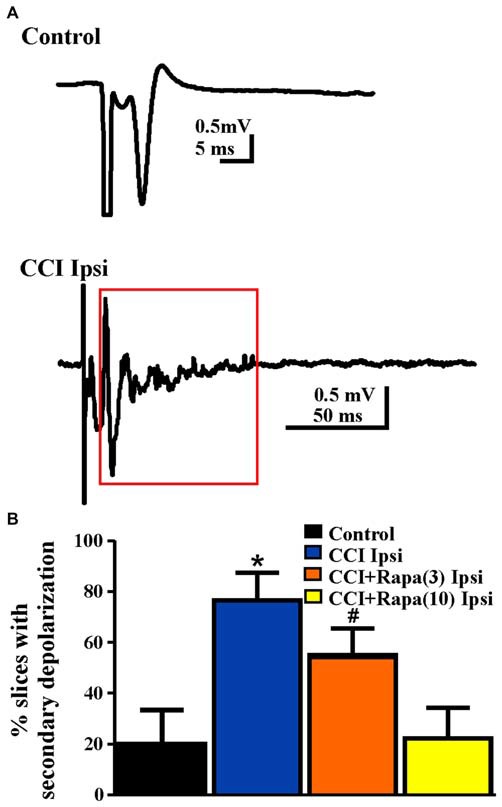
**Network excitability of dentate granule cells after antidromic electrical stimulation 8–13 weeks post-injury. (A)** Representative traces of field potential responses to antidromic hilar stimulation in control and CCI-injured mice. A secondary depolarization was often observed in slices ipsilateral to CCI injury (red box indicates area of secondary depolarization). **(B)** Percentage of slices ipsilateral to injury that displayed secondary depolarization from control, CCI Ipsi, CCI + Rapa(3) Ipsi, and CCI + Rapa(10) Ipsi treatment groups. Error bars represent SEM; *p < 0.05 for CCI Ipsi relative to control or CCI + Rapa(10). ^#^Indicates *p* < 0.05 for CCI + Rapa(3) Ipsi vs. control.

Increased network excitability (Hunt et al., 2009) and synaptic connectivity between granule cells (Hunt et al., 2010) emerge in the dentate gyrus several weeks after CCI injury. Antidromically-evoked field potentials following electrical stimulation of the hilus were examined in slices perfused with nominally Mg^2+^ free ACSF with bicuculline (30 μM) 8–13 weeks after injury. In these recordings, a single antidromic population spike was elicited after hilar stimulation in most slices (29/36) from five control animals (Table 1; Figure 5). In contrast, a secondary after discharge was observed in most slices (11/14 slices from 9 mice) from the ipsilateral hemisphere of vehicle-treated, CCI-injured mice (Figure 5), similar to previous findings at the same time point post-injury in this model (Hunt et al., 2009; Chi-square statistic = 15.295; *p* < 0.0001; Figure 5B). Low-dose rapamycin treatment reduced, but did not normalize the percentage of slices with secondary depolarization in the ipsilateral hemisphere (5/9 slices from 8 mice; Chi square statistic = 1.3707; *p* = 0.241 vs. CCI ipsilateral; Figure 5B). In mice treated with high-dose rapamycin, the percentage of slices ipsilateral to the injury that responded with a secondary depolarization (2/10 slices from 10 mice) was significantly lower than for vehicle-treated CCI-injured mice (Chi-square statistic = 8.0607; *p* = 0.0045; Figure 5B), and was similar to controls (*p* = 0.9687). Notably, 86% of slices from CCI-injured, rapamycin-treated mice that displayed increased network excitability also exhibited localized mossy fiber sprouting near the recording site upon *post hoc* examination. Overall, rapamycin treatment after CCI significantly reduced dentate granule cell network excitability following hilar antidromic stimulation, but excitability was maintained in slices with mossy fiber sprouting.

### sEPSCs in DGCs

**Figure 6:**
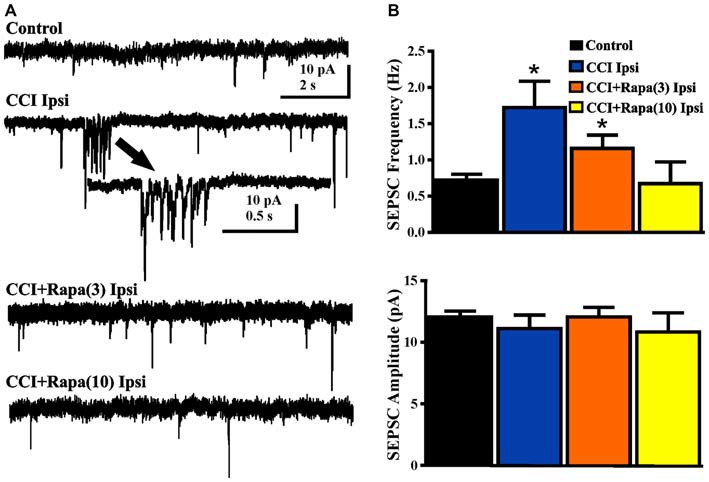
**Spontaneous EPSCs (sEPSCs) in dentate granule cells 8–13 weeks post-injury. (A)** Representative traces of sEPSCs in dentate granule cells ipsilateral to injury from four treatment groups: sham-operated control, CCI injury + vehicle (CCI Ipsi), CCI injury + 3 mg/kg rapamycin (CCI + Rapa(3)), and CCI injury + 10 mg/kg rapamycin (CCI + Rapa(10)). All recordings were performed in the presence of nominally Mg^2+^-free ACSF containing 30 μM bicuculline. Arrow indicates expanded example of a burst of sEPSCs in a dentate granule cell from the vehicle-treated CCI group. **(B)** Mean sEPSC frequency and amplitude in the same treatment groups. Error bars indicate SEM; *p < 0.05.

Spontaneous EPSCs (sEPSCs) were recorded from dentate granule cells in slices from vehicle- and rapamycin-treated mice 8–13 weeks after CCI. Slices were perfused with nominally Mg^2+^ -free ACSF containing 30 μM bicuculline and cells were voltage-clamped at −70 mV (Figure 6A). sEPSC frequency was greater in DGCs ipsilateral to CCI injury relative to controls (control: 0.72 ± 0.08 Hz; *n* = 29, CCI: 1.51 ± 0.38 Hz, *n* = 14 cells from 7 mice; *F*_(3,56)_ = 5.336, *p* = 0.0026; One Way ANOVA, Tukey’s; Figure 6B). The increase in sEPSC frequency after CCI injury was reduced in rapamycin-treated mice (CCI + rapamycin 3 mg/kg: 1.16 ± 0.18 Hz; *n* = 11 cells from 9 mice; *p* = 0.187 vs. CCI + vehicle; CCI + rapamycin 10 mg/kg: 0.67 ± 0.30 Hz; *n* = 12 cells from 7 mice; *p* = 0.0267 vs. CCI + vehicle). Relative to controls, however, sEPSC frequency in dentate granule cells from rapamycin-treated (3 mg/kg) mice remained significantly elevated (*p* = 0.0154), with no difference between controls and rapamycin treated (10 mg/kg) mice (*p* = 0.847). No differences in sEPSC amplitude were found between any of the experimental groups (Control: 12.04 ± 0.49 pA; CCI + vehicle: 11.12 ± 1.09 pA; CCI + rapamycin 3 mg/kg: 12.06 ± 0.78 pA; CCI + rapamycin 10 mg/kg: 10.84 ± 1.54 pA; *p* = 0.6894; One Way ANOVA, Tukey’s; Figure 6B). Increased sEPSC frequency was observed ipsilateral to the injury after CCI. Low-dose rapamycin treatment reduced, but did not eliminate this increase, whereas sEPSC frequency was similar to controls in mice treated with 10 mg/kg rapamycin daily.

## Discussion

Multiple outcome measures associated with epileptogenesis after CCI have been established in the dentate gyrus, allowing for mechanistic investigation of cellular events subsequent to TBI. Within a few weeks after CCI trauma in mice, sprouting of dentate granule cell axons to proximal granule cell dendrites in the inner molecular layer of the dentate gyrus (i.e., mossy fiber sprouting) occurs, synaptic reorganization of dentate granule cells is observed near the injury site, and mice develop spontaneous seizures after several weeks (Hunt et al., 2009, 2010; Guo et al., 2013). This study focused on the role of mTOR signaling in PTE development using the CCI model of TBI. Previous studies on the effects of rapamycin treatment in acquired epilepsy models have focused mainly on the anatomical phenotype of mossy fiber sprouting and the functional correlation with seizure frequency (Buckmaster and Lew, 2011; Guo et al., 2013). However, increased mossy fiber sprouting and seizure frequency were noted after cessation of rapamycin treatment, suggesting epileptogenic mechanisms that trigger mTOR activity and subsequent neurogenesis or other cellular activity post-injury may be sustained, although the activity itself is suppressed during rapamycin treatment. To avoid confounds associated with reemergence of mTOR activity-related phenotypes after cessation of rapamycin treatment, we continued rapamycin treatment daily throughout the duration of our experiments. Here, we report that rapamycin treatment after CCI injury inhibits the progression of epileptogenesis after focal brain injury in a manner that involves effects on several cellular outcomes associated with development of spontaneous seizures after TBI, including post-injury neurogenesis, mossy fiber sprouting, and synaptic reorganization in the dentate gyrus ipsilateral to the injury. No differences were observed contralateral to injury, implying rapamycin alone had little effect compared to uninjured controls. Both doses of daily rapamycin treatment (3 and 10 mg/kg) were effective in reducing the proportion of mice that developed spontaneous seizures, consistent with effects of an intermediate dose (6 mg/kg) administered for four weeks after CCI injury (Guo et al., 2013). Both rapamycin doses also significantly reduced post-injury neurogenesis in the granule cell layer. Other outcomes, including mossy fiber sprouting and elevated synaptic excitation were reduced, but not abolished by the lower rapamycin dose, but were abrogated by the high-dose regimen. Granule cell layer area and FJB staining measurements indicated that neither rapamycin dose significantly reduced overall neuronal death after CCI, but cell death was reduced by the high dose regimen at a specific site along the septo-temporal hippocampal axis. The lack of effect on cell death is consistent with the reemergence of spontaneous seizures after cessation of rapamycin treatment in previous reports and suggests that important components of the underlying injury that triggers the eventual development of epilepsy are not abrogated by mTOR inhibition, even though several other cellular correlates of epileptogenesis are suppressed during high-dose rapamycin treatment.

### Newborn Neurons

The continual adult generation of select neuron populations, including within the subgranular zone (SGZ) of the hippocampus (Altman and Das, 1965; Eriksson et al., 1998), remains one of the least well understood types of experience-dependent brain plasticity. Adult neurogenesis has been proposed to either decrease (Gould and Tanapat, 1997; Rola et al., 2006) or increase after TBI (Liu et al., 1998; Parent et al., 1998; Dash et al., 2001; Arvidsson et al., 2002; Chirumamilla et al., 2002), and markers of adult neurogenesis were diminished if rapamycin was administered prior to pilocarpine-induced status epilepticus (Zeng et al., 2009), suggesting effects of rapamycin on proliferation and/or survival of newborn neurons. Two weeks post-injury, we identified an increase in DCX-positive cell density in the dentate gyrus ipsilateral to the injury, consistent with previous reports linking seizures with increased adult neurogenesis (Parent et al., 1997, 1998, 2006).

Both rapamycin doses used in this study suppressed post-injury neurogenesis in association with diminished seizure prevalence, consistent with the hypothesis that mTOR inhibition is associated with decreased adult neurogenesis. Little is known about the role immature dentate granule cells play in the functional connectivity of hippocampal circuitry after brain injury, but several studies have linked seizure-associated synaptic reorganization to abnormal connectivity of newborn neurons. The hypothesis that newborn dentate granule cells contribute selectively to synaptic reorganization and epileptogenesis has been proposed (Kron et al., 2010). Genetic enhancement of the PI3K→AKT→mTOR pathway by deletion of PTEN (i.e., transgenic phosphatase and tensin homolog), specifically in neural progenitors, is sufficient to increase adult neurogenesis (Amiri et al., 2012) and cause development of spontaneous seizures (Pun et al., 2012; Hester and Danzer, 2013; Lasarge et al., 2015). Further, mTOR inhibition with rapamycin attenuates development of seizures in PTEN knockout mice (Sunnen et al., 2011), implicating the mTOR-mediated modulation of adult neurogenesis in the development of acquired epilepsy. Interestingly, and perhaps paradoxically, increased mTOR activation has been proposed as a means of diminishing injury and improving cognitive recovery after TBI in patients (Don et al., 2012), whereas use of rapamycin to suppress mTOR activity post-TBI has been proposed to prevent or suppress epileptogenesis (Guo et al., 2013). A better understanding of the contribution of newly-born neurons to adult brain function in healthy and disease states appears necessary in order to optimally utilize mTOR modulation after TBI for cognitive recovery and prevention of PTE.

### Mossy Fiber Sprouting

Synaptic reorganization in the dentate gyrus after CCI injury was also reduced by rapamycin treatment. The relationship between modulation of post-injury synaptic reorganization and reducing the prevalence of spontaneous seizures in rapamycin treated mice is unclear, since the treatment did not completely eliminate either seizures or mossy fiber sprouting. This was most apparent at the low dose of rapamycin, where both mossy fiber sprouting and seizure prevalence were reduced but not eliminated. It is possible that even limited synaptic reorganization is sufficient for seizure expression (Hunt et al., 2010; Pun et al., 2012). The general failure of many studies to quantitatively link post-injury mossy fiber sprouting with spontaneous seizures, along with recent studies showing that seizures develop in the absence of robust mossy fiber sprouting in the pilocarpine-induced status epilepticus model of epilepsy after rapamycin treatment, have led to the suggestion that mossy fiber sprouting and spontaneous seizures are not causally linked. However, even when quantitatively reduced in rapamycin treated mice, some degree of mossy fiber sprouting was observed in all mice that displayed spontaneous seizures here. While no proven causal relationship exists to date, the qualitative presence of post-injury mossy fiber sprouting suggests it cannot be excluded as a cellular correlate of epileptogenesis. Alternatively, mossy fiber sprouting and synaptic reorganization may represent a secondary change associated with epileptogenesis, since rapamycin prevented significant mossy fiber sprouting, but not spontaneous seizures, in rapamycin treated mice after pilocarpine-induced status epilepticus (Heng et al., 2013). It is also possible that the synaptically reorganized dentate gyrus reflects a relatively mature stage of epileptogenic circuit formation, whereas other factors that occur in earlier stages of epileptogenesis, including cell loss and adult neurogenesis, contribute to the eventual change in connectivity. The cellular triggers of epileptogenesis remain poorly-defined. However, the association of rapamycin treatment with a reduction in seizure prevalence and cellular markers of PTE is consistent with the hypothesis that activation of the mTOR pathway plays a role in development of PTE.

Mice receiving severe unilateral CCI injury begin to develop PTE after a latent period of ~6–10 weeks post-injury (Hunt et al., 2009; Guo et al., 2013). The percentage of mice reported to develop spontaneous behavioral or electrographically measured seizures varies from 36–50% (Hunt et al., 2009; Guo et al., 2013). Here 40% of mice receiving CCI without drug treatment developed spontaneous behavioral seizures, similar to previous reports. Rapamycin treatment (6 mg/kg) for 4 weeks after injury reduced seizure prevalence, with 13% of mice expressing electrographically identified seizures (Guo et al., 2013). The proportion of mice exhibiting spontaneous behavioral seizures observed here was similarly reduced in mice that received either low- or high-dose rapamycin treatment in the present study to 8 and 9%, respectively, representing a trend toward reduced seizure prevalence. Notably, our behavioral seizure measurements probably underestimate the total number of seizures in all groups, due to periodic observation. Rapamycin treatment therefore tended to reduce, but did not eliminate, development of generalized spontaneous seizures after CCI.

### Network Excitability

Electrophysiological indices of network excitability are increased in the dentate gyrus after CCI, including evoked network responses and sEPSC frequency in dentate granule cells. Commensurate with diminished mossy fiber sprouting, synaptic excitability was suppressed in rapamycin treated CCI-injured mice, and was comparable to controls with the high dose regimen, although increased network excitability was observed in slices where mossy fiber sprouting was present. Others have reported effects of mTOR inhibition on mossy fiber sprouting, but assessment of synaptic or network activity has not been reported previously. Increased electrophysiological responses are hallmarks of synaptic reorganization in excitatory circuitry of the dentate gyrus and are correlated with mossy fiber sprouting and development of spontaneous seizures in this and other epilepsy models (Dudek and Spitz, 1997; Patrylo and Dudek, 1998; Lynch and Sutula, 2000; Winokur et al., 2004; Hunt et al., 2009, 2010, 2011, 2012). Axon plasticity after injury or seizures is a feature of many neuron types, and these neurons could also contribute to increased sEPSC frequency in dentate granule cells. Although a causative link between synaptic reorganization and epilepsy remains controversial, these results are consistent with reduced functional synaptic reorganization after CCI injury in rapamycin-treated mice.

### Cell Death

Another hallmark of CCI injury is selective cell loss, particularly in the hilus and dentate gyrus (Hicks et al., 1993; Graham et al., 2000; Maxwell et al., 2003; Anderson et al., 2005). The use of FJB as a marker to infer cell degeneration and necrotic cell death indicates peak cell loss within the first 3 days after CCI injury, with a gradual reduction in neuronal degeneration over time (Anderson et al., 2005; Ansari et al., 2008; Hall et al., 2008). Rapamycin was shown previously to reduce FJB staining in dentate gyrus, CA3, and CA1 regions of the hippocampus three days post-injury at a site ~1 mm posterior to epicenter (Guo et al., 2013). Here, we assessed the full septo-temporal axis of the hippocampus and found that neither low- nor high-dose rapamycin treatment attenuated FJB staining overall in the dentate gyrus and hilus. Although FJB labeling remained significantly greater than in controls, however, 10 mg/kg rapamycin treatment did attenuate FJB staining relative to vehicle treatment after CCI in the ipsilateral hemisphere in the same area (i.e., ~ 1 mm from injury epicenter) as previously reported (Guo et al., 2013). This region corresponds to the area of greatest cell death in this brain injury model. Together, these results suggest the possibility that rapamycin may moderately suppress post-injury neuronal death regionally, even if cell death overall in the dentate gyrus and hilus is unaffected by the treatment.

## Conclusion

The findings of this study are consistent with the hypothesis that mTOR inhibition reduces synaptic reorganization among granule cells and inhibits post-traumatic epileptogenesis after CCI. Continuous rapamycin treatment reduced the percentage of mice expressing spontaneous seizures, inhibited measures of synaptic reorganization in the granule cell layer, and abrogated the increase in neurogenesis following CCI injury. Notably, even the highest dose of rapamycin failed to completely prevent PTE or specific cellular changes associated with epileptogenesis, including post-injury cell death, in a substantial number of injured mice. These findings suggest that mTOR inhibition alters disease progression, but may not prevent the initiation of epileptogenesis. The relationship between adult neurogenesis, excitatory synaptogenesis, and seizure susceptibility remains uncertain in the CCI and other models of acquired epilepsy, but we hypothesize that the inhibition of post-injury neurogenesis is a significant feature of the anti-epileptogenic effects of rapamycin treatment following CCI injury. Effects of hormones and growth factors that cross the blood brain barrier after injury have been attributed to an increase in neurogenesis mediated by mTOR activity, and several studies have targeted this mechanism as a therapeutic option to restore cognitive function post-injury (Lu et al., 2005; Sun et al., 2009; Xiong et al., 2012; Carlson et al., 2014). However, the present results imply that potential benefits of increased mTOR signaling might be mitigated by the potentially detrimental epileptogenic effects over time. This study highlights the need for further work to understand how newly born dentate granule cells integrate and function in the injured hippocampus and how this integration is related to both functional recovery after TBI and the potentially increased risk of seizure susceptibility. Understanding mTOR’s role in these processes may help define the critical features of epileptogenesis and recovery from TBI.

## Author Contributions

CB, JB, and BS made substantial contributions to the conception and design of the work; acquisition and analysis were performed by CB and JB; interpretation of data was done by CB, JB, and BS. CB, JB, and BS drafted and revised the manuscript critically for important intellectual content. All authors approve the version of the manuscript to be published and agree to be accountable for all aspects of the work in ensuring that questions related to the accuracy or integrity of any part of the work are appropriately investigated and resolved.

## Conflict of Interest Statement

The authors declare that the research was conducted in the absence of any commercial or financial relationships that could be construed as a potential conflict of interest.
